# Characterization of a transcriptome from a non-model organism, *Cladonia rangiferina*, the grey reindeer lichen, using high-throughput next generation sequencing and EST sequence data

**DOI:** 10.1186/1471-2164-13-575

**Published:** 2012-10-30

**Authors:** Sini Junttila, Stephen Rudd

**Affiliations:** 1 Turku Centre for Biotechnology, University of Turku and Åbo Akademi University, Tykistökatu 6, 20520, Turku, Finland

**Keywords:** Non-model organism, *Cladonia rangiferina*, Transcriptome sequencing, Functional annotation

## Abstract

**Background:**

Lichens are symbiotic organisms that have a remarkable ability to survive in some of the most extreme terrestrial climates on earth. Lichens can endure frequent desiccation and wetting cycles and are able to survive in a dehydrated molecular dormant state for decades at a time. Genetic resources have been established in lichen species for the study of molecular systematics and their taxonomic classification. No lichen species have been characterised yet using genomics and the molecular mechanisms underlying the lichen symbiosis and the fundamentals of desiccation tolerance remain undescribed. We report the characterisation of a transcriptome of the grey reindeer lichen, *Cladonia rangiferina*, using high-throughput next-generation transcriptome sequencing and traditional Sanger EST sequencing data.

**Results:**

Altogether 243,729 high quality sequence reads were de novo assembled into 16,204 contigs and 49,587 singletons. The genome of origin for the sequences produced was predicted using Eclat with sequences derived from the axenically grown symbiotic partners used as training sequences for the classification model. 62.8% of the sequences were classified as being of fungal origin while the remaining 37.2% were predicted as being of algal origin. The assembled sequences were annotated by BLASTX comparison against a non-redundant protein sequence database with 34.4% of the sequences having a BLAST match. 29.3% of the sequences had a Gene Ontology term match and 27.9% of the sequences had a domain or structural match following an InterPro search. 60 KEGG pathways with more than 10 associated sequences were identified.

**Conclusions:**

Our results present a first transcriptome sequencing and de novo assembly for a lichen species and describe the ongoing molecular processes and the most active pathways in *C. rangiferina*. This brings a meaningful contribution to publicly available lichen sequence information. These data provide a first glimpse into the molecular nature of the lichen symbiosis and characterise the transcriptional space of this remarkable organism. These data will also enable further studies aimed at deciphering the genetic mechanisms behind lichen desiccation tolerance.

## Background

Lichen is formed through a symbiotic relationship between a fungus and a photosynthetic partner, which can be either an alga or a cyanobacterium [1]. The fungus, or mycobiont, forms a three-dimensional vegetative structure called a thallus, in which the photosynthetic partners, or photobionts, are located. The thallus is a complex and undifferentiated body with upper and lower surfaces of densely aggregated fungal hyphae. The algal (or cyanobacterial) cells are surrounded by fungal hyphae and are maintained beneath the upper cortex. The thallus structure enables gas exchange for the photobiont population and competes for well-illuminated space above the ground [2]. Lichen thalli are not individuals but are instead genetically heterogeneous consortia of an unknown number of participants [2]. It has however been calculated that the algal cells comprise only approximately 7% of the total thallus volume [3].

One-fifth of fungal species form obligate symbiotic associations with green alga or cyanobacteria [4]. This increases to about 46% for ascomycete fungi. The processes of lichenisation and its physiology are therefore relevant to the understanding of ascomycete relationships and the evolution of mechanisms for the control and maintenance of plant-fungal interactions. The number of algal species that can participate within lichenisation processes is less broad. An estimated 100 species from 40 genera have been reported to form lichen symbioses [5].

The molecular nature of the lichen symbiosis remains debated. Some researchers have observed a controlled parasitism of the photobiont by the mycobiont [1]. Others have reported a mutualistic relationship [4]. Regardless of the nature of the relationship, lichens inhabit some of the harshest terrestrial climates on earth, and have demonstrated a capacity to survive even the most challenging environmental extremes of outer space [6]. Most lichen species are tolerant to profound desiccation. This is facilitated by the adoption of an anhydrobiotic state, and in some cases, e.g. *Lobaria pulmonaria*, morphological adaptations to limit the harmful effects of photoinhibition [7]. The anhydrobiotic state is facilitated through the accumulation of specific metabolites and polysaccharides that limit the damage caused by desiccation and maintain sufficient physiological integrity so that any resulting damage can be repaired upon eventual rewetting [8,9].

Damage caused by reactive oxygen species (ROS) is one of the key threats to surviving anhydrobiosis [10]. The ROS protective mechanisms are not yet characterised, although basic roles for antioxidants in lichen desiccation and rehydration have been demonstrated in a variety of lichen species [9,11-14]. The accumulation of antioxidant and photo-protective compounds during desiccation plays an important role in the rapid reestablishment of metabolism and photosynthesis following rehydration [15,16]. A lichen can better tolerate desiccation than either fungus or alga alone since its combined antioxidant and photo-protective mechanisms are more effective than those of the isolated partners [11]. Understanding and characterising the molecular mechanisms that enable lichens to survive years of desiccation, and the approaches to photo-protection and ROS control have potential applied value in the development of innovative strategies for trait improvement in higher plants.

There are many uncharacterised molecular mechanisms that appear specific to lichen, but there are few resources available that can facilitate the genetic characterisation of these processes. Genetic resources have however been established for the systematic study and classification of lichens, reviewed in [5]. These resources are biased to the needs of taxonomists and evolutionary researchers and are not suitable for functional genomics. Research on lichen gene expression is currently limited to a single publication [17] and unpublished data from Messen & Ott, and as yet there is little in the way of either high-throughput genome sequence or expressed sequence tag (EST) data available for any lichen species. As queried on 7^th^ of February 2012, there were 1,864 lichen EST sequences in GenBank. The whole genome sequence of the lichen *Xanthoria parietina* has been completed but not yet published according to the DOE Joint Genome Institute and a genomic survey of the algal symbiont of the lichen *Ramalina farinacea* is available in the Short Read Archive of the NCBI.

The emergence of the next generation of DNA sequencing technologies has enabled the transcriptome and genome sequencing of numerous non-model organisms. For non-model organisms, de novo genome assembly of short read data is complicated but many transcriptomes have been sequenced from non-model species and published over the last years [18-22]. The annotation process remains challenging, especially for species with no close relatives with a sequenced reference genome.

Our objective was to generate a lichen transcriptome using both next-generation sequencing and traditional Sanger sequencing. The Roche GS FLX platform was used for the next-generation sequencing. Sanger sequencing was performed to complement the FLX run data with its long sequence reads. We produced additional Sanger EST sequences from axenically grown symbiotic partners (*C. rangiferina* and *Asterochloris* sp.) to train classification models for predicting the genome of origin of lichen sequences. We have obtained a basic view of the ongoing molecular processes and have identified the most active biological pathways in *C. rangiferina*. Our transcriptome data brings an increase to the amount of publicly available lichen sequences and provides a starting point for further studies into lichen functional genomics.

## Results

### Sequencing and de novo assembly

**Figure 1:**
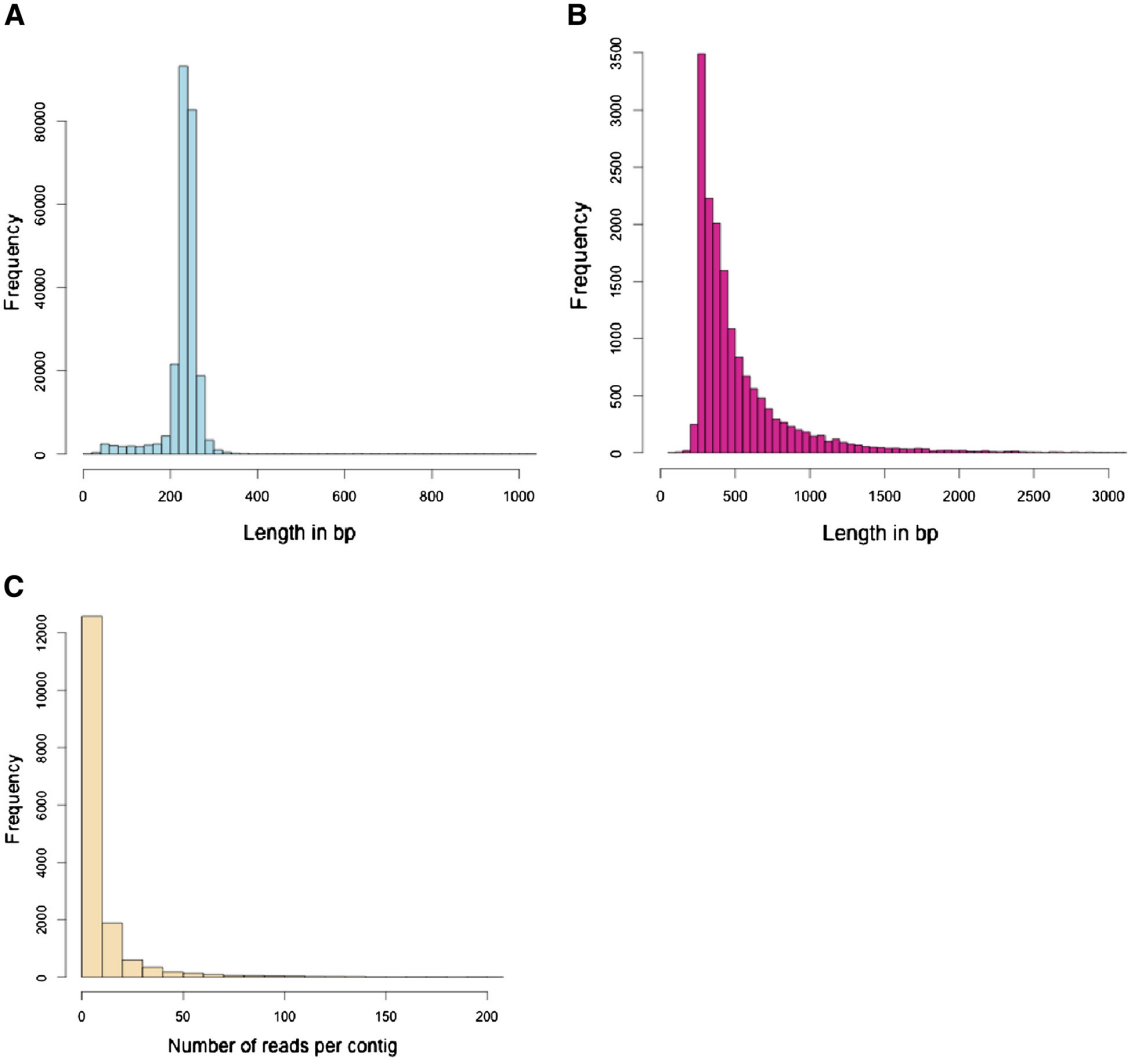
**The statistics of the reads and assembled contigs.A** The distribution of the lengths of all sequences obtained from both GS FLX high-throughput sequencing run and traditional Sanger sequencing, **B** the distribution of contig lengths after de novo assembly, and **C** the distribution of reads per contig.

Lichen transcriptome sequences were generated using both a high-throughput next-generation sequencing technology run and traditional Sanger sequencing from lichen cDNA libraries. The GS FLX run yielded 240,990 sequence reads (55.8 Mbp) and 2,990 EST reads were obtained by Sanger sequencing. The average length of the cDNA sequence reads was 232 bp and 723 bp for GS FLX sequences and Sanger sequences, respectively (Table 1). The majority of the GS FLX reads were shorter than 400 bp (Figure 1A), although the maximum read length was 1,258 bp. After quality and length trimming in the assembly software 243,729 high quality reads were included in the de novo assembly and they were assembled into 16,204 contigs and 49,587 singletons. The GS FLX reads have been submitted to the Short Read Archive [SRA:SRA050676] and the high quality Sanger sequences have been submitted to GenBank [GenBank:JK811361-JK813924 and GenBank:GH717691-GH717859]. A list of the contig and singleton sequences is available as Additional file 1. The length of the contigs varied between 87 and 5,426 bp (Figure 1B). The N50 value of the assembled contigs was 569 bp, and the average length of the contigs was 528 bp (Table 2). 79.7% of the reads were assembled into contigs while 20.3% of the reads remained as singletons. The sequence reads contained within contigs varied between 2 and 1,435 with the average number of reads per contig being 12 (Figure 1C). The coverage of the contigs varied between 1 and 588 with the average coverage being 3.9.

**Table 1:** The statistics of the reads

	**All reads**	**GS FLX reads**	**Sanger reads**
No of reads	243 980	240 990	2990
Maximum length	1258	509	1258
Minimum length	5	7	5
Average length	232	232	723
Median length	238	238	715

**Table 2:** The statistics of the contigs assembled from the reads

**No of contigs**	**16 204**
Maximum length	5426
Minimum length	87
Average length	528
Median length	403
Maximum number of reads	1435
Minimum number of reads	2
Average number of reads	12
Median number of reads	5
N50	569

### Annotation and classification of the contigs and singletons

**Figure 2:**
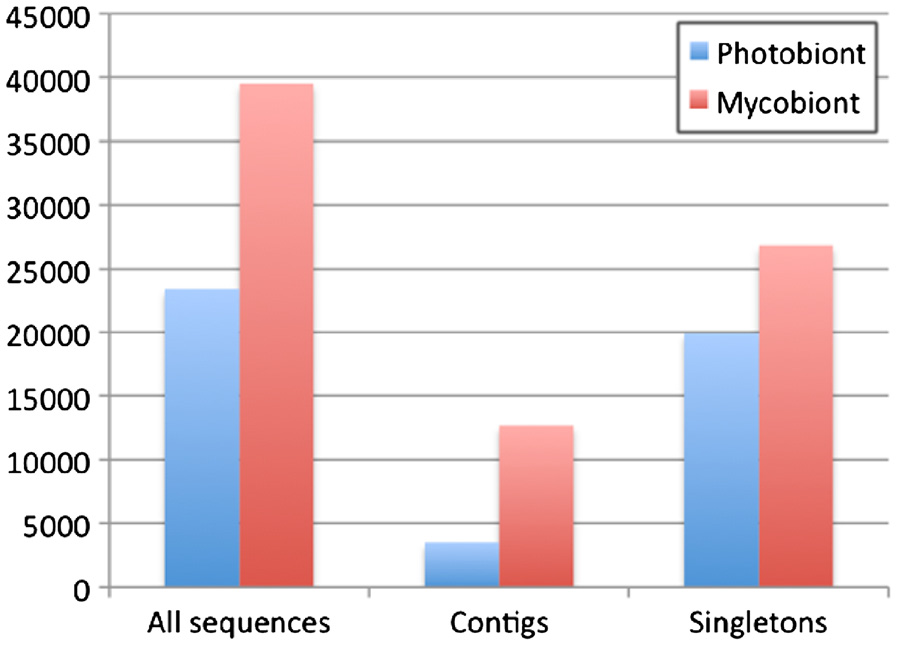
**The division of the sequences into fungal and algal origin.** The division of the sequences into fungal and algal origin according to Eclat. The results of the Eclat analysis are shown separately for all sequences, contigs and singletons.

As lichen is a symbiosis of two distinct organisms the genome of origin was predicted for the assembled contigs and singletons to define whether sequences originated from the mycobiont (fungus) or the photobiont (alga) genomes. This classification was performed using Eclat [23]. cDNA sequences produced from axenically grown symbiotic partners (*C. rangiferina* and *Asterochloris* sp.) were used to train the required Eclat models to classify and discriminate between the two different genomes. Altogether 1,009 fungal and 854 algal sequences derived from the axenically grown symbiont cDNA libraries were used for the training. The minimum sequence length for the classification was set at 100 bp. 2,829 lichen sequences could not be classified due to their insufficient sequence length. The complete set of contigs and singletons was classified and 62.8% of the sequences were predicted as mycobiont sequences and 37.2% as photobiont sequences (Figure 2, Table 3). When classifying only the contigs, 78.4% were predicted as mycobiont and 21.6% as photobiont. For singleton sequences alone 57.4% mycobiont and 42.6% photobiont sequences, were predicted.

**Table 3:** The division of the sequences into algal and fungal origin by Eclat

	**Photobiont**	**Mycobiont**
All sequences	23 406 (37.2%)	39 551 (62.8%)
Contigs	3492 (21.6%)	12 712 (78.4%)
Singletons	19 914 (42.6%)	26 840 (57.4%)

**Figure 3:**
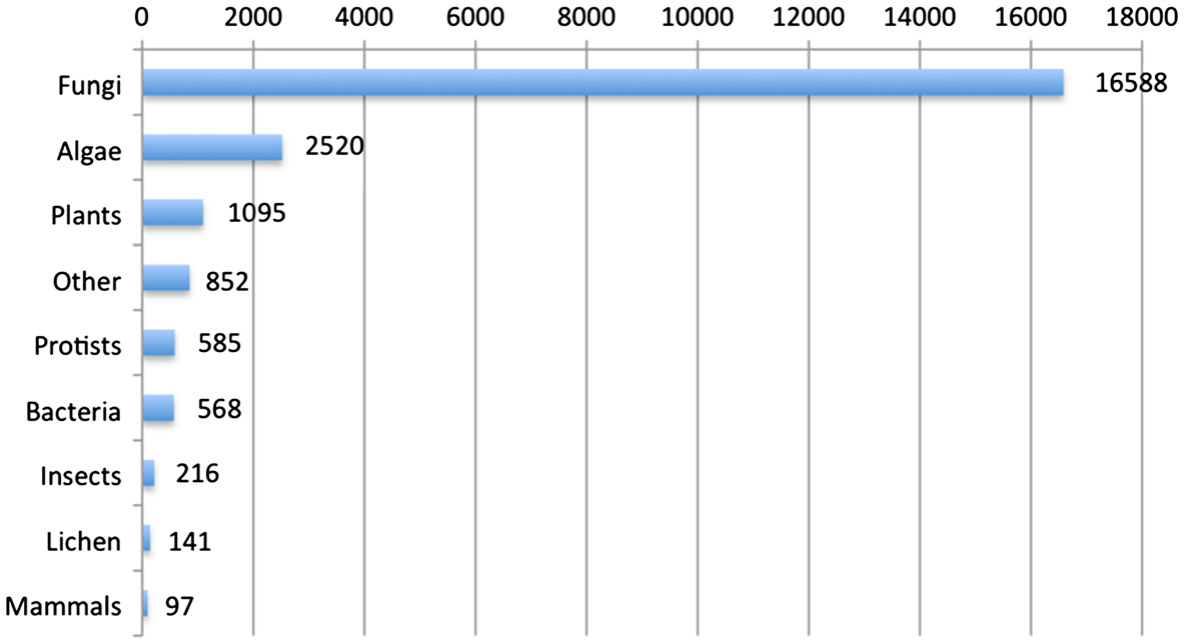
**The taxonomic distribution of the top BLAST hits.** The best BLAST hits for each sequence were divided into nine categories: algae, fungi, plants, bacteria, protist, mammals, insects, lichen and other. The number at the end of the bar indicates the number of sequences.

The sequences were annotated using the Blast2GO software [24]. The contigs and singletons were included in the annotation process. The annotations included best BLASTX match selection, Gene Ontology ID assignment, enzyme code assignments and InterPro domains calculation. BLASTX analysis using the NCBI non-redundant (nr) database revealed that 22,662 sequences (34.4%) had a match in the database with a cut-off value of 1e-10. For the contigs alone, the percentage of sequences with a BLAST result was 57.2%. 73.2% (16,588 sequences) of the sequences had the best BLAST match to a fungal species, while 11.1% (2,520 sequences) had an algal species as the best match (Figure 3). 4.8% (1,095 sequences) had the best match to a plant species, 2.5% (568 sequences) to a bacterium, 2.6% (585 sequences) to a protist, 1% (216 sequences) to an insect, 0.6% (141 sequences) to a lichen, 0.4% (97 sequences) to a mammal and 3.8% (852 sequences) to other. 19,250 sequences (29.3 %) had matches to Gene Ontology (GO) term annotations and 18,324 sequences (27.9 %) had InterPro matches.

**Figure 4:**
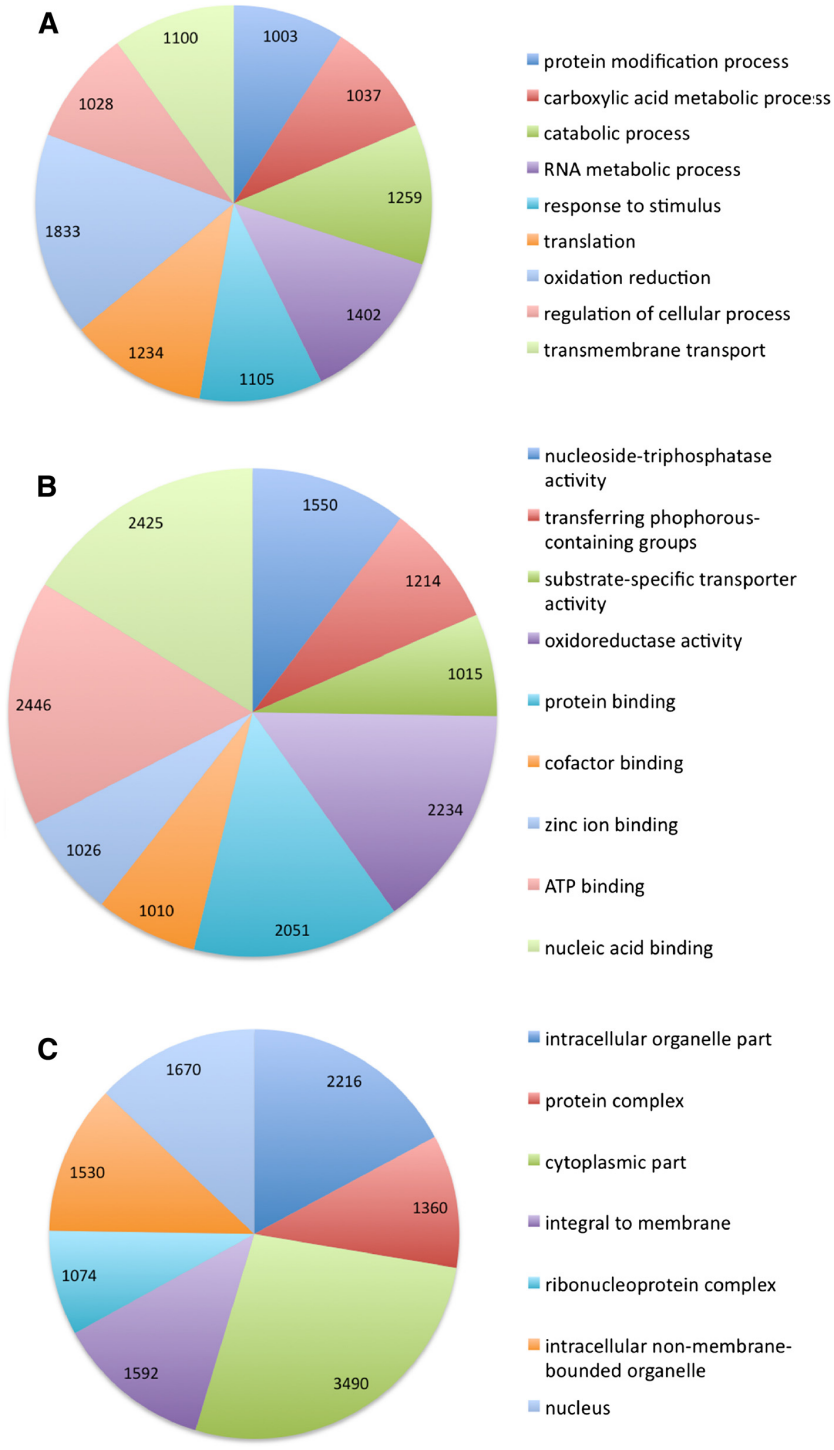
**GO term distribution of the sequences.** GO terms with the most associated sequences for **A** biological process category, **B** cellular component category, and **C** molecular function category. The numbers in the charts indicate the number of sequences associated with the particulate GO term.

The GO annotations of the sequences form three core categories; biological process (BP), cellular component (CC) and molecular function (MF). 22,736 sequences were assigned to biological process, 13,086 to cellular component and 29,170 to molecular function. There were 2,209 unique BP terms, 576 unique CC terms and 1,528 unique MF terms assigned to the sequences. The GO terms with the largest number of assigned sequences in the BP category (Figure 4A) were oxidation reduction (1,833 sequences), RNA metabolic process (1,402 sequences), catabolic process (1,259 sequences), translation (1,234 sequences) and response to stimulus (1,105 sequences). For CC (Figure 4B) the terms with the most sequences were cytoplasmic part (3,490 sequences), intracellular organelle part (2,216 sequences) and nucleus (1,670 sequences). In MF category (Figure 4C) the terms with the most sequences were ATP binding (2,446 sequences), nucleic acid binding (2,425 sequences), oxidoreductase activity (2,234 sequences), protein binding (2,051 sequences) and nucleoside-triphosphatase activity (1,550 sequences). The sequences were also assigned to KEGG pathways (Table 4) and the pathways with the most sequences were purine metabolism (696 sequences), methane metabolism (239 sequences), pyrimidine metabolism (218 sequences), thiamine metabolism (209 sequences) and oxidative phosphorylation (203 sequences). Altogether 60 KEGG pathways were identified and the full pathway list is available as Additional file 2.

**Table 4:** The top 20 KEGG pathways

**Pathway**	**No of sequences in pathway**	**No of identified enzymes**
Purine metabolism	696	47
Methane metabolism	239	27
Pyrimidine metabolism	218	25
Thiamine metabolism	209	6
Oxidative phosphorylation	203	9
Pyruvate metabolism	194	26
Glycolysis/Gluconeogenesis	187	25
Arginine and proline metabolism	182	37
Aminoacyl-tRNA biosynthesis	180	21
Propanoate metabolism	167	14
Carbon fixation pathways in prokaryotes	164	19
Amino sugar and nucleotide sugar metabolism	158	27
Citrate cycle (TCA cycle)	156	19
Alanine, aspartate and glutamate metabolism	152	27
Valine, leucine and isoleucine degradation	146	18
Fatty acid metabolism	138	17
Glycine, serine and threonine metabolism	132	26
Tryptophan metabolism	128	14
Starch and sucrose metabolism	124	32
Glutathione metabolism	120	16

The top 20 KEGG pathways with the most associated sequences. The full KEGG pathway table is provided as Additional file 2.

## Discussion

Lichens are symbiotic organisms consisting of two components; a fungal partner or mycobiont and an algal partner or photobiont. Lichens are remarkable organisms in their ability to tolerate extreme environmental conditions including even outer space [6,25,26]. They resume photosynthesis rapidly after even long periods of desiccation [14]. The molecular mechanisms underlying lichen’s survival adaptations are uncharacterised and genomic resources for lichens are limited. To gain a glimpse of the molecular nature of these neglected organisms, we have generated EST sequences from grey reindeer lichen, *Cladonia rangiferina*, using both high-throughput next-generation sequencing and traditional Sanger sequencing. The sequences were de novo assembled with 79.7% of the reads assembling into contigs, and only 20.3% of the reads remaining as singletons. These values are similar to other non-model organism transcriptome de novo assemblies [27,28].

As the grey reindeer lichen is a symbiotic organism comprising of two distinct genomes, the *Asterochloris* genome and the *Cladonia rangiferina* genome, the sequences were classified to identify their genome of origin, and to obtain an estimate of the ratio of fungal to algal sequences in the lichen transcriptome. More than half of the sequences were classified as fungal sequences, although this varied depending on whether contigs or singletons were classified. The difference between contigs and singletons is in both their length and cis-substantiation suggesting that sequence length affects classification. BLASTX best match taxonomic assignment shows a similar ratio of fungus to alga / plant sequences ratio as the predictive classification performed by Eclat. This suggests that the taxonomic assignment performance is consistent and robust. Although probable non-target sequences, as evidenced by BLASTX analysis, may be present within our sequence collection, the amount of contamination is modest and should not affect the classification. The ratio of the two organisms has been estimated previously with 7% of cells being of algal origin [3]. Similar values were obtained in an analysis of *Lobaria pulmonaria* protein spectra, where 10% of the spectra were assigned to green algal proteins [29]. Our results suggest a higher percentage of algal transcripts expressed in wetted lichen tissue. Transcript abundance likely correlates most with transcriptional activity and with thallus cell abundance to a lesser level . However, our results appear to confirm the mycobiont as the dominant partner in the symbiosis even in the context of gene expression.

Since no lichen reference genome has yet been published annotation was performed by comparing homologous protein sequences with BLASTX. 57.2% of all assembled contig sequences had a BLAST hit when run against the non-redundant protein sequence database. A considerable fraction of the sequences remain as unidentified and apparently novel sequences. This percentage was considerably lower for the singleton sequences. Similar homology results with lower BLAST match percentages for singletons have been reported for other non-model organism transcriptome de novo assemblies [30]. The numbers are also concordant with those published by Joneson et al. who found a significant homology to 50% of *Cladonia grayi* sequences in the nr database [17]. Some of the sequences without a BLAST match are likely UTRs, but novel, lichen specific sequences are also likely present in this sequence collection. The cDNA libraries used for sequencing were also un-normalized and therefore there can be a significant redundancy in the ESTs sequenced. In addition, as no reference genome is yet available for any lichen species, the reads were not mapped - this would yield an ideal assignment.

A significant majority of the sequences had either an alga, a fungus, or a lichen species as the best match in the BLAST search (Figure 3). However, only 0.6% of the sequences had the best match to a lichen species, which illustrates the current lack of lichen sequences in the public databases. The largest non-target taxonomic groups were the bacteria (2.5% of sequences), protists (2.6% of sequences), and other (3.8%). Since lichen thalli are also known to contain internal bacterial communities [31], the presence of bacterial sequences from the lichen microbiome is not unexpected.

To decipher the biological meaning of the BLAST annotated sequences, GO and KEGG databases were used for functional annotation, while InterPro search was performed to identify recognisable protein motifs within our sequence collection. Lichens have been found to protect themselves from the damage caused by ROS during desiccation by using antioxidants [11,32] but the enzymatic antioxidants are also involved in removing ROS produced during normal metabolism [33]. This could be reflected by the GO terms related to oxidation within the most enriched GO terms (Figure 4). Also in the identified KEGG pathways (Table 4, Additional file 2), glutathione metabolism pathway potentially indicates that constitutive protection mechanisms against ROS are active in wetted lichen thallus, as has been previously studied by measuring high amounts of reduced glutathione in undesiccated lichens [9]. These results support the hypothesis that highly-desiccation tolerant lichens rely mainly on constitutive protection mechanisms, which require constant levels of gene expression [33].

Several enriched GO terms and most of the identified KEGG pathways were involved in energy, nucleotide and amino acid metabolisms. These findings are consistent with earlier results, in which spectra assigned to proteins involved in post-translational modifications, energy production and conversion were highly abundant in the mycobiont [29]. The same study found that proteins involved in energy production and conversion strongly dominate the protein fraction of green alga. Similarly, pathways involved in photosynthesis (carbon fixation in photosynthetic organisms, porphyrine and chlorophyll metabolism) are among the KEGG pathways with highest amount of sequences in our results.

The carbohydrate produced by the photobiont is leaked and taken up by the mycobiont and consequently converted to arabitol and mannitol through the phosphate pentose pathway [34]. The transport-related enriched GO terms and the pentose phosphate pathway within the identified KEGG pathways potentially indicate that this mechanism is active in the studied lichen thallus. Surprisingly, methane metabolism had the second highest amount of sequences within the KEGG pathways. The sequences associated with this pathway could potentially be novel, lichen-specific sequences, which have a high homology to the proteins associated with methane metabolism, but which are in reality associated with an uncharacterised pathway, e.g. the production of a lichen-specific secondary metabolite. 27.9% of the sequences had a match in the InterPro database, and this suggests that although a reasonable proportion of the sequences contain a number of recognisable protein motifs, there are many unrecognisable sequences, some of which may contain novel protein structures.

## Conclusions

We have sequenced the transcriptome of a non-model organism, grey reindeer lichen, through high-throughput next-generation sequencing and traditional Sanger sequencing from cDNA libraries. Lichen is a symbiotic relationship between a fungus and an alga and therefore also the transcriptome is comprised of genes originating from the two distinct genomes. We were able to discern the genome of origin for the lichen sequences by using sequences derived from axenically cultured symbiotic partners as training sequences. Often the bottleneck of analysing sequence material from non-model organisms lies in the annotation process, as no reference genome is available, and also the sequences available in public databases can be almost non-existent. Using information from several different databases we have described here the first de novo assembly and characterization of any lichen transcriptome. The results give a preliminary glimpse into the molecular nature of the lichen symbiosis and the transcriptional space of this resilient organism as we have identified KEGG pathways and GO terms associated with the lichen sequences. These data will also significantly increase the amount of publicly available lichen sequences. We will be exploring the lichen gene expression further in our ongoing research by designing a custom microarray based on these sequence data and comparing the differences in gene expression between differently treated lichen samples. These results are expected to give more insight to lichen desiccation mechanisms and reveal genes involved in the rapid re-establishment of photosynthesis upon hydration.

## Methods

### Lichen collection, culturing of axenic fungus and alga and their identification

Lichen was collected from the island of Kuusisto, in Kaarina, Finland. It was cleaned and stored in desiccated state at −20 C. Axenic cultures of the symbiotic partners *C. rangiferina* and *Asterochloris* sp. were prepared using the modified Yamamoto method [35] and cultured at 21°C. *C. rangiferina* was cultured on Malt Yeast Extract [35] agar plates and *Asterochloris* sp. on Organic Nutrient Medium for Trebouxia [36] agar plates. The identity of the cultures was confirmed by sequencing the ITS regions using ITS1F and ITS4 primers for *C. rangiferina* and ITS1T and ITS4T primers for *Asterochloris* sp. [37,38]. The DNA for the sequencing was extracted with Qiagen’s DNeasy Plant Mini Kit (Qiagen, Germany) according to manufacturer’s instructions. The ITS region was amplified using the primers and sequenced with ABI Prism 3130xl Genetic Analyzer capillary DNA sequencer following a BigDye v3.1 (Applied Biosystems, USA) labelling reaction.

### RNA extraction

Prior to RNA extraction the lichen tissue was weighed and rewetted with tap water overnight. Fungal and algal tissues were collected off the agar plates. All tissue types were powdered in liquid nitrogen using mortar and pestle. The total RNA extraction was performed as previously described [39], and mRNA was isolated from the total RNA with Nucleo-Trap mRNA kit (Macherey-Nagel, Duren, Germany).

### EST sequencing

mRNA extracted from lichen thallus and axenically grown fungal and algal symbionts were used to construct phage cDNA libraries using the ZAP-cDNA Gigapack III Gold Cloning (#200450) cDNA library synthesis kit (Stratagene, La Jolla, USA) according to manufacturer’s instructions. Size fractionation was achieved using gel electrophoresis. Gel slices corresponding of between 500–1000 bp and 1000–3000 bp in size were excised and gel-purified cDNA was cloned into a phage library. cDNA library clones were sequenced on an ABI PRISM 3130xl Genetic Analyzer capillary DNA sequencer following a BigDye v3.1 labelling reaction.

### Next-generation sequencing

The double-stranded cDNA for the next-generation sequencing was prepared using SuperScript Double-Stranded cDNA Synthesis kit (Invitrogen, Life Technologies, USA) according to manufacturer’s instructions. Invitrogen’s Oligo dT_(12–18)_ primer was used in the synthesis reaction. The concentration of the cDNA was measured with NanoDrop and altogether 5.12 μg of double-stranded cDNA was used as starting material for the Roche GS FLX sequencing. The high-throughput sequencing was performed at the DNA Sequencing and Genomics Laboratory at the Institute of Biotechnology at University of Helsinki, Helsinki, Finland according to previously described methods [40]. The cDNA was amplified with Phi29 polymerase (GenomePhi, GE Healthcare, USA) and a single-stranded sequencing library was created according to the FLX instructions. The GS FLX run data were filtered with the GS Run Browser and reads not passing the quality filters were removed from the data.

### Bioinformatic analyses

The sequences were de novo assembled with CLC Genomics Workbench software version 4.9 (CLCBio, Denmark). Prior to the assembly the sequences were trimmed in the CLC Genomics Workbench. All of the sequences were trimmed using quality scores with a limit of 0.05, in addition the adapter sequences potentially present at either end of the GS FLX sequences were removed, and the Sanger sequences were compared against the NCBI UniVec database to remove vector and polylinker sequences. Sequences shorter than 15bp were removed from the analysis. In the de novo assembly the minimum contig length was set to 250 bp and voting method was chosen as the conflict resolution parameter. The reads were then mapped back to the contigs. The N50 value of the assembly was calculated by summarizing the lengths of the biggest contigs until 50% of the total contig length was reached. The minimum contig length in this set is the number that was used to report the N50 value of the de novo assembly.

Eclat [23] was used to identify the genome of origin for the contigs and singletons derived from lichen tissue. The Sanger sequences obtained from the axenically grown algal and fungal symbiont cDNA libraries were used to train Eclat and build a model file for the classification. Base calling for the sequences used in Eclat training was performed using Phred [41] and the training sequences were compared against a modified NCBI UniVec database using cross_match to identify vector and polylinker sequence substrings prior to use in Eclat. The minimum sequence length for classification was set at 100 bp.

Blast2GO [24] tool was used for BLASTX, GO term and enzyme code annotation, Interpro scans and KEGG pathway analysis of the contigs and singletons. BLASTX [42] was used to compare the assembled contigs and singletons to a non-redundant (nr) protein sequence database from the NCBI GenBank database [43]. BLASTX matches were filtered using an arbitrary cut-off of 1e-10. Combined graphs were produced from the GO annotation results and the most enriched GO terms were visualized in multilevel format. GO terms with less than 1,000 sequence matches were not included in the analysis. Interpro scan was performed against all of the available InterPro databases. KEGG pathway information was retrieved based on the enzyme code annotation.

## Competing interests

The authors declare that they have no competing interests.

## Authors’ contributions

SJ and SR planned the experiments, SJ carried out all of the experiments and did the data analysis. SR helped with the data analysis. Both authors wrote the manuscript. Both authors read and approved the final manuscript.

## Supplementary Material

Additional file 1 **Cladonia Rangiferina Contigs And Single tons.fasta.zip.** The contig and singleton sequences from the de novo assembly in compressed fasta format. Click here for file

Additional file 2 **KEGGPathways.xlsx.** The identified KEGG pathways associated with the sequences. Click here for file
